# Sensitivity and Contrast Characterization of PMMA 950K Resist Under 30 keV Focused Ga^+^ Ion Beam Exposure

**DOI:** 10.3390/mi16080958

**Published:** 2025-08-20

**Authors:** Mukhit Muratov, Yana Shabelnikova, Sergey Zaitsev, Renata Nemkayeva, Nazim Guseinov

**Affiliations:** 1 Department of Physics and Technology, Al-Farabi Kazakh National University, 050040 Almaty, Kazakhstan; mukhit.muratov@gmail.com (M.M.); quasisensus@mail.ru (R.N.); 2 Institute of Microelectronics Technology and High Purity Materials, Russian Academy of Sciences, Chernogolovka, 142432 Moscow, Russia; janeshabeln@yandex.ru (Y.S.); bachokg@gmail.com (S.Z.)

**Keywords:** ion-beam lithography, PMMA 950K, Ga^+^ FIB, sensitivity, contrast, energy deposition profile

## Abstract

In this study, the key lithographic performance of PMMA 950K resist was evaluated by exposure to a 30 keV focused gallium (Ga^+^) ion beam. The sensitivity and contrast of PMMA 950K were directly compared with those of electron exposure under identical development conditions. It was found that the sensitivity of PMMA 950K to Ga^+^ ions for 50 nm films reaches a value of about 0.4 μC/cm^2^, which is more than 250 times higher than its sensitivity to electron exposure. A method for evaluating the resist contrast during ion exposure is proposed in this work, taking into account the highly non-uniform dose distribution across the resist depth; it yielded a contrast value of γ = 2.6, which is consistent with the result obtained with electron exposure (γ = 2.8). In addition, a pronounced dependence of the resist sensitivity on the resist thickness was found: with an increase in thickness from 10 nm to 60 nm the sensitivity decreases by an order of magnitude. The obtained results form a reliable methodological basis for characterizing the behavior of polymer resists under ion irradiation and provide valuable recommendations for optimizing lithography with a focused beam of Ga^+^ ions when creating nanostructures for microelectronics, photonics, and quantum technologies.

## 1. Introduction

Modern nanotechnology is rapidly developing towards the miniaturization of functional components used in micro- and nanoelectronics, photonics, sensors, plasmonics, quantum optics, and biomedical engineering [1,2,3]. This trend, reflected in strategic roadmaps such as the International Roadmap for Devices and Systems (IRDS), envisages the development of devices with dimensions of less than 10 nm, including quantum dots, plasmonic resonators, next-generation biosensors, and highly integrated circuits [4,5]. The implementation of such structures places strict demands on lithographic technologies, including a resolution of less than 10 nm, high accuracy of pattern formation, stability, and reproducibility of processes [6,7]. Electron beam lithography (EBL) remains one of the most versatile nanostructuring tools due to its ability to form complex two-dimensional and three-dimensional structures with nanometer resolution [8,9]. However, wider industrial application of EBL is hampered by both physical and technological limitations. Among the physical limitations, the main one is the proximity effect caused by backscattered and secondary electrons, which cause unwanted exposure in adjacent areas, reducing the patterning accuracy [10,11]. From the technological side, EBL is characterized by low throughput due to its sequential writing nature and high doses required for exposure (typically 10–500 μC/cm^2^), which leads to long processing times and increased energy consumption [12,13]. For example, exposing an area of about 1 cm^2^ can take from several hours to a full day depending on the process conditions, making EBL unsuitable for mass production [14]. Focused ion beam lithography (FIBL), especially using gallium (Ga^+^) ions emitted from liquid metal ion sources (LMISs), is emerging as a promising alternative to EBL [15,16]. Due to the high mass of Ga^+^ ions their angular scattering in solids is minimal and their trajectories are almost linear, which at ion energies on the order 30 keV leads to energy deposition predominantly in the near-surface region of the resist, usually within a few tens of nanometers [17,18]. In addition, the linear energy transfer (LET) of ions in resist materials is significantly higher than that of electrons, which ensures increased sensitivity—the required exposure doses can be 2–3 orders of magnitude lower than in the case of EBL [19]. An additional advantage of FIBL is the almost complete absence of the proximity effect. Secondary and backscattered ions have an extremely short range, and the resulting secondary electrons have low energy: backscattered electrons are virtually absent. As a result, exposure occurs almost exclusively due to the main ion beam, with minimal parasitic effects, which ensures high spatial resolution and selectivity of pattern formation. Modern dual-beam systems (FIB-SEM), which combine electron and ion columns, are capable of generating Ga^+^ ion beams with diameters below 10 nm and energies up to 30 keV. These systems are becoming powerful tools for high-precision nanofabrication, including prototyping, mask fabrication, material modification, and quantum and functional materials research [20,21,22]. Despite these advantages, the widespread adoption of Ga^+^-FIB in lithographic processes requires a deeper understanding of the ion–resist interactions. In particular, the behavior of poly (methyl methacrylate) (PMMA) 950K resist, widely used in EBL due to its well-characterized sensitivity, high contrast, and stability, remains understudied in the case of ion exposure. Although the sensitivity of PMMA is lower than that of specialized negative resists such as HSQ and positive resists such as ZEP (10–50 μC/cm^2^), it has high technological flexibility, chemical stability, and pronounced dose-dependent development characteristics [23]. However, key lithographic parameters such as the sensitivity (D_0_) and contrast (γ) of PMMA under ion irradiation have not yet been systematically quantified either experimentally or by modeling.

Unlike EBL, where electron energy is deposited at depths of up to 1 μm, under ion irradiation energy deposition is limited to a thin near-surface layer, forming a sharp energy density gradient [24]. This fundamentally changes the kinetics of radiation-induced reactions such as chain scission and cross-linking. In particular, PMMA 950K exhibits a positive tone at doses below ~0.5 μC/cm^2^ and a negative tone at doses above ~1 μC/cm^2^ due to the cross-linking of macromolecules. This dose-dependent transition is observed for both electron and ion irradiation, indicating common radiation–chemical mechanisms. Such dual-tone behavior opens up opportunities for fabricating gradient, multilayered, or grayscale nanostructures. However, the successful implementation of these applications relies on precise control of exposure conditions and a quantitative understanding of lithographic characteristics under ion beams. In particular, reliable data on sensitivity, contrast, and depth profiles of deposited energy are essential for predicting feature dimensions and optimizing process parameters, especially considering the limited penetration depth of heavy ions.

The goal of this study is to quantitatively evaluate the key lithographic performance of PMMA 950K resist exposed to a 30 keV focused Ga^+^ ion beam. Particular attention is paid to determining sensitivity and contrast and comparing them with typical values obtained using electron beam lithography (EBL). Sensitivity is defined as the minimum dose required to develop the resist down to the substrate. The contrast under Ga^+^ ion exposure is quantified using a model that takes into account the depth distribution of the absorbed energy, providing a rigorous and physically sound interpretation of the lithographic response of PMMA 950K resist. The depth distribution of the deposited energy is described by a shifted Gaussian distribution. For this purpose, the residual resist layer thickness is measured experimentally as a function of the exposure dose. The obtained data serve as a basis for the optimization and standardization of ion lithography processes using PMMA 950K, facilitating their integration into scalable nanofabrication technologies.

## 2. Materials and Methods

Standard single-crystal silicon wafers with a diameter of 100 mm and a thickness of 500 μm were used as substrates for resist deposition by the spin coating method. Before resist deposition, the wafers were subjected to sequential ultrasonic cleaning in acetone (purity ≥ 99.99%) and isopropyl alcohol (purity ≥ 99.8%) for 10 min each, after which they were dried at 200 °C for 5 min on a hot plate to remove residual moisture. Commercially available polymethyl methacrylate (PMMA) with a molecular weight of 950 K was used as a resist in two concentrations designated as A4 and A2, corresponding to solutions in anisole with a concentration of 4 wt.% and 2 wt.%, respectively. The PMMA 950K resist was purchased from MicroChem (Kayaku Advanced Materials, Westborough, MA, USA). The resist layer was formed by spin coating using an SCK-200 setup (Instras Scientific, Ridgefield Park, NJ, USA). Two different resist thicknesses were obtained: about 50 nm when applying solution A2 at 6500 rpm for 30 s and about 180 nm when applying solution A4 at 3000 rpm for 30 s. Post-deposition soft baking was conducted on a calibrated 5 inch magnetic hotplate (FOUR E’S SCIENTIFIC, Guangzhou, China) at 180 °C for 3 min to remove residual solvent and ensure uniform film densification. Electron and ion beam exposures were performed on a Quanta 3D 200i Dual Beam (FEI Company, Hillsboro, OR, USA) equipped with a NanoMaker lithography control system (Interface company, Moscow, Russia), which provides both design and exposure control. A test template in the form of a dose wedge was used to characterize the lithographic response, in particular sensitivity and contrast, of the PMMA resist. The structure was a linear array of rectangles sequentially exposed at linearly increasing doses: 10 × 3 μm^2^ for electron beam lithography (EBL) and 10 × 5.5 μm^2^ for focused ion beam lithography (FIB). For electron beam lithography (EBL), the dose ranged from 0 to 10,000 μC/cm^2^ with a minimum step of 5 μC/cm^2^. The acceleration voltage was 30 keV and the beam current was 30 pA. For focused ion beam lithography (FIB) of 30 keV Ga^+^ ions, the dose ranged from 0 to 10 μC/cm^2^ with a minimum step of 0.04 μC/cm^2^. The ion beam current was 10 pA. Development after exposure was carried out in a solution of methyl isobutyl ketone (MIBK, purity ≥ 99.0%) and isopropyl alcohol (IPA, purity ≥ 99.8%) in a ratio of 1:3 (*v*/*v*) for 8 s at room temperature (25 °C). Development was stopped by rinsing the samples in deionized water for 5–10 s, after which the samples were dried with nitrogen gas flow. Characterization of the surface topography and resist profile was carried out using an atomic force microscope (AFM) Solver Spectrum (NT-MDT, Zelenograd, Russia) in tapping mode at a scan rate of 0.1 Hz to improve the clarity of the boundaries and vertical resolution. NSG01 cantilevers (TipsNano, Tallinn, Estonia) with a tip radius of less than 10 nm, a nominal spring constant of 1.45–15.1 N/m, and a resonance frequency of about 170 kHz were used. The obtained AFM data were processed using the Nova Px 3.2.5 software to extract height profiles and quantitatively analyze the formed resist structures. The AFM-measured profiles of the dose wedge were linearly converted into the corresponding exposure dose values (Supplementary Materials). The visualization of the developed dose wedges was performed using a Leica DM6000 M optical microscope (Leica Microsystems, Wetzlar, Germany) equipped with a bright-field reflected light mode, with objective lenses ranging from 20× to 100×. All experiments were conducted in clean laboratory rooms, although not certified according to ISO cleanroom standards. The TRIM module from the SRIM-2013 package was used for modeling (www.srim.org). All calculations were performed for gallium ions (Ga^+^) with an energy of 30 keV incident on a PMMA polymer resist with a density of 1.18 g/cm^3^ and a chemical composition of C_5_H_8_O_2_. The Detailed Calculation with Full Damage Cascades mode was used in the calculations; the number of ions was 20,000 to ensure statistical reliability [25,26].

## 3. Results and Discussion

### 3.1. Sensitivity of PMMA 950K to Ion and Electron Beam Exposure

**Figure 1:**
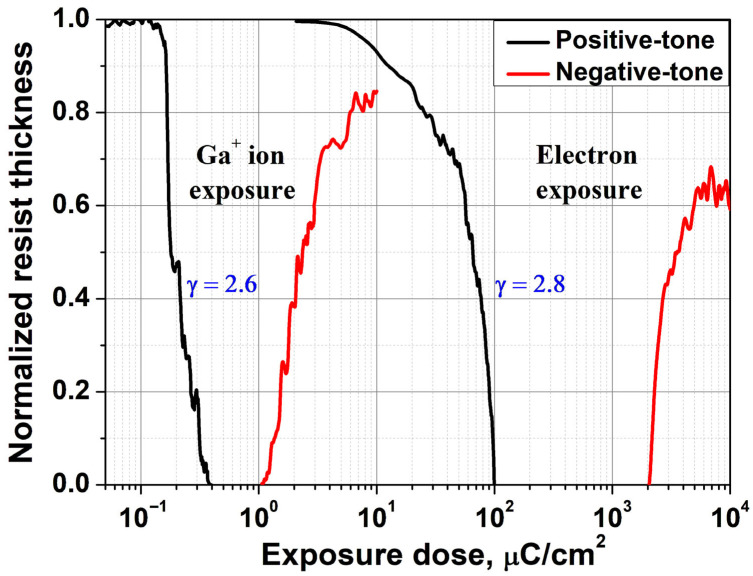
Comparison of the dose response characteristics of PMMA 950K resist under Ga^+^ ion and electron beam exposure (30 keV particle energy, 50 nm resist thickness).

The first stage of the study focused on the quantitative evaluation of the PMMA 950K resist sensitivity under gallium (Ga^+^) ion beam exposure and the comparison of the obtained values with the results typical for electron beam lithography (EBL) performed at the same particle energy of 30 keV. To ensure a correct comparison of the lithographic parameters, a thin resist film of about 50 nm thick was used. This choice was based on the TRIM evaluation results, according to which the average penetration depth of Ga^+^ ions at a given energy in PMMA with a density of 1.18 g/cm^3^ is about 42.6 nm. This thickness ensures absorption of most of the beam energy inside the resist volume with a small penetration of ions into the substrate. In contrast, with electron beam exposure a significant portion of the energy is transferred into the substrate due to the greater electron penetration depth, resulting in a reduction in the deposited energy density available to effectively break polymer chains in the thin resist layer. Figure 1 presents the dose-dependent normalized residual thickness of the resist after ion and electron beam exposure (detailed AFM thickness measurement data are provided in the Supplementary Materials). Both samples were developed simultaneously under identical conditions to ensure a consistent comparison of their lithographic responses.

As shown in Figure 1, the sensitivity of PMMA 950K under Ga^+^ ion exposure is significantly higher than that under electron beam exposure. Under otherwise identical conditions, the dose required for complete removal of the resist layer is approximately 0.4 μC/cm^2^ in the ion exposure case, which is about 250 times lower than the corresponding value of ~100 μC/cm^2^ in EBL. This indicates the high efficiency of localized energy deposition by Ga^+^ ions as most of their energy is dissipated within the resist volume, efficiently initiating scission of PMMA macromolecules.

At higher doses, PMMA 950K undergoes a tone reversal from positive to negative under both ion and electron exposure, due to cross-linking of polymer chains. This transition is accompanied by the formation of a denser, carbon-rich network and the release of volatile products (H_2_, CO, CO_2_), leading to shrinkage of the resist and reduction in residual film thickness [23]. Table 1 summarizes the experimentally determined dose values corresponding to full dissolution in the positive-tone regime and to the onset of negative-tone behavior (50% residual thickness) for both exposure types.

The contrast of the resist was quantitatively determined in the electron exposure case by analyzing the slope of the linear portion of the dissolution curve in logarithmic scale [27], yielding a value of γ = 2.8, consistent with values reported in the literature [28]. In the case of ion exposure, this graphical approach is not applicable due to the highly non-uniform energy deposition profile with depth. A quantitative evaluation of contrast under ion exposure was performed in the following section using a model that accounts for the depth distribution of absorbed energy, providing a rigorous and physically grounded interpretation of the lithographic response of the PMMA 950K resist under Ga^+^ ion beam exposure.

### 3.2. Depth Profile Analysis in Thick PMMA 950K Films Exposed to Ga^+^ Ions

**Figure 2:**
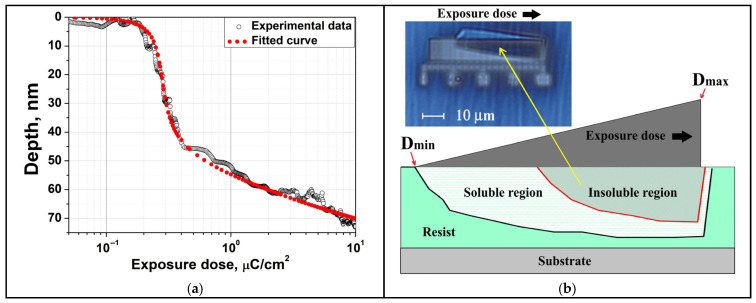
(**a**) Experimental development depth versus dose for PMMA 950K exposed to Ga^+^ ions and corresponding model fit based on absorbed energy distribution. (**b**) Schematic of spatial resist solubility distribution induced by the non-uniform energy profile under ion exposure, with an inset showing an optical microscopy image of the cross-linked region.

At the second stage of the study, a quantitative analysis of the development depth profile was performed for a thick PMMA 950K resist layer with a thickness of 180 nm, exposed to a beam of Ga^+^ ions with an energy of 30 keV in a given dose range. The use of a thick resist is due to the need to ensure complete absorption of ion energy within the polymer volume, since at a given thickness the ion penetration depth completely fits into the resist layer. The experimental dependence of development depth on dose is shown in Figure 2a. The observed dependence of the development depth versus dose indicates a strongly non-uniform energy deposition profile within the resist.

To interpret the data, a model was employed that links the development rate to the degree of polymer chain scission. In positive-tone resists, solubility in the developer is governed by radiation-induced breaking of macromolecular chains [29,30]. A decrease in average molecular weight leads to a reduced entanglement of polymer chains and a corresponding increase in dissolution rate. The following power-law relation [30,31,32] empirically describes this behavior:

(1) $$V\;=\;aM^{-\gamma }$$

where V is the development rate, M is the average molecular weight, γ is the resist contrast, and a is an empirical constant.

Assuming the degree of chain scission is directly proportional to the local absorbed energy density ε, the following relation is established [10,33]:

(2) $$\frac{V(z)}{V_{0}}\;=\;{\left(\frac{\varepsilon (z)}{\varepsilon_{0}}\right)}^{\gamma }$$

where *V*_0_ is the development rate at a reference dose, *ε*_0_ is the corresponding energy density, and γ is the contrast parameter.

Since nearly all of the ion beam energy is consumed in breaking polymer chains, the local absorbed energy density can be expressed as ε(z) ∝ D·εₚ(z) where D is the exposure dose, and εₚ(z) is the normalized energy deposition profile with depth z. The latter was modeled using a shifted Gaussian function, as proposed in [24]:

(3) $$\varepsilon_{p}(z)\;=\;A\exp\left(-\frac{{\left(z\;-\;B\right)}^{2}}{2C^{2}}\right)$$

with B = 0.47 L, C = 0.38 L, where L is the mean free path length of ions, and *A* is a normalization constant. The relationship between the development depth *h* and dose *D* is then given by the integral expression:

(4) $$\int_{0}^{h}\frac{dz}{{\left(\varepsilon_{p}(z)\right)}^{\gamma }}\;=\;{\left(\frac{D}{D_{0}}\right)}^{\gamma }$$

where *D*_0_ is a fitting parameter incorporating *V*_0_, *ε*_0_, and normalization constants of εₚ(z).

As a result of measured depth–dose dependence approximation calculated in accordance with (4) curves, the value contrast γ = 2.6 was revealed (Figure 2a) for TRIM calculated value L = 42.6 nm and D_0_ = 0.0158 μC/cm^2^. The residual function or Normalized Error Value (NEV) was explored to quantify the deviation between the experimental and modeled profiles. It was calculated as integral squared deviation of calculated points from measured, normalized on integral from squared measured profile. It should be noted that for the fitting problem specified by (4) the NEV function has a shallow minimum and variations in γ have little effect. So a twofold NEV increase (from its minimal value 0.15% to 0.3%) corresponds with a wide range of contrast values from 1.5 to 3. The obtained contrast value differs only slightly from the result of our experiment with electron exposure γ = 2.8 and is within the range of literature data for PMMA 950K exposed to electrons and light ions γ = 2–12, which is due to variations in molecular weight, developer, and deposition and development conditions [28,34,35,36]. Due to the pronounced non-uniformity of the deposited dose with depth, and considering that sensitivity is defined as the minimum exposure dose required to fully develop the resist down to the substrate, Figure 2a clearly illustrates a significant dependence of sensitivity on resist thickness. Specifically, when applying a resist layer with a thickness of approximately 10 nm, the sensitivity is about 0.2 μC/cm^2^, whereas at a thickness of around 60 nm it decreases, reaching approximately 2 μC/cm^2^. Figure 2b presents a schematic illustration of the resist behavior under a dose gradient. It highlights the depth profile of absorbed energy density and delineates two principal zones: an insoluble region formed at high doses due to polymer cross-linking and a partially soluble zone resulting from molecular chain scission near the interface with the main resist body, enabling the lift-off of the cross-linked segment. This spatial development behavior was further corroborated by the inset optical micrograph shown in Figure 2b, acquired after development of the dose wedge in the developer, which captures the moment when the cross-linked region has detached from the substrate but has not yet been completely removed—visually confirming the predicted separation between dissolution and cross-linking zones and underscoring the inherently three-dimensional nature of the process.

## 4. Conclusions

A direct comparative study of the lithographic characteristics of the positive tone of a PMMA 950K resist exposed to Ga^+^ ions and 30 keV electrons and developed under the same conditions was carried out. It was found that the sensitivity to Ga^+^ ions for a 50 nm thick resist layer is approximately 0.4 μC/cm^2^, which is more than 250 times higher than the sensitivity to electron exposure. This significant increase is explained by the strong localization of the deposited energy under heavy ion exposure. At high doses for both types of exposure cross-linking processes and a change in tone were observed, confirming the universality of the chemical transformation mechanism in PMMA for both types of exposure. To estimate the lithographic parameters under conditions of highly non-uniform energy distribution over the depth, an analytical model based on the depth–exposure dose dependence h(D) was developed. The obtained contrast value was γ = 2.6, which is in good agreement with the value γ = 2.8 obtained with electron exposure using the classical approach. The proposed method is a reliable tool for both contrast analysis and indirect assessment of ion braking characteristics in polymer resists. A pronounced dependence of sensitivity on the resist thickness was also revealed: with an increase in thickness from 10 nm to 60 nm the sensitivity decreases by an order of magnitude. Despite this, the sensitivity to ions remains significantly higher than to electron exposure.

The obtained results form a reliable methodological basis for optimizing lithography processes with a focused beam of Ga^+^ ions and expand their applicability in the field of high-precision nanofabrication for the needs of microelectronics, photonics, and quantum technologies. The proposed technique serves as a universal platform for analyzing the behavior of resists under the influence of heavy ions and contributes to the development of scalable, high-precision lithographic processes.

## Figures and Tables

**Table 1:** Experimental dose values for transition of PMMA 950K between positive- and negative-tone regimes (50 nm thickness, 30 keV).

Exposure Type	Positive-Tone, [μC/cm^2^]	Negative-Tone, [μC/cm^2^]
Ga^+^ ions	~0.4	~2.4
Electrons	~100	~2600

## Data Availability

All data is included in the manuscript.

## Supplementary Materials

The following supporting information can be downloaded at: https://www.mdpi.com/article/10.3390/mi16080958/s1, Table S1. Parameters of the dose wedges used for sensitivity and tone behavior analysis under 30 keV Ga^+^ ion exposure. Figure S1. Typical dose wedges #2 and #3 used to characterize positive- and negative-tone behavior of a 50 nm PMMA 950K resist layer on a silicon substrate under 30 keV Ga^+^ ion exposure. Table S2. Parameters of the dose wedges used for electron beam exposure to evaluate resist sensitivity, contrast, and tone behavior. Figure S2. Construction of the full depth–dose profile in a 180 nm PMMA 950K resist layer on a silicon substrate under 30 keV Ga^+^ ion exposure: (a) AFM profile of wedge #4; (b) dose conversion; (c) merged wedges #1–#4; (d) final stitched curve.
