# Supplementary material for: Sensitivity and Contrast Characterization of PMMA 950K Resist Under 30 keV Focused Ga+ Ion Beam Exposure
Source: Micromachines (Basel). 2025 Aug 20;16(8):958. doi: 10.3390/mi16080958 (PMC12388081; doi:10.3390/mi16080958)
Supplement: Supplementary file 1 [file micromachines-16-00958-s001.zip › micromachines-3742609-supplementary.pdf]

Supplementary Material

Sensitivity and Contrast Characterization of PMMA 950K Resist under 30 keV Focused Ga^+ Ion Beam Exposure

Mukhit Muratov[1], Yana Shabelnikova[2], Sergey Zaitsev[2], Renata Nemkayeva[1] and Nazim Guseinov [1,*]

[1]Al-Farabi Kazakh National University, 050040, Almaty, Kazakhstan
[2]Institute of Microelectronics Technology and High Purity Materials RAS, 142432 Chernogolovka, Moscow region, Russia
*Correspondence: solar_neo@mail.ru

Introduction

The dose curve represents the dependence of the residual resist thickness on the exposure dose and allows the determination of two key lithographic parameters characteristic of conventional electron beam lithography: the resist sensitivity — defined as the minimum dose required to completely remove the resist down to the substrate (in the case of positive-tone behavior), and the contrast — which characterizes the steepness of the transition between exposed and unexposed regions. In the case of negative-tone behavior, the onset dose of cross-linking is also determined, which typically corresponds to 50% of the residual resist thickness.

To obtain the dose curve, a test structure in the form of a dose wedge is used, through which the exposure dose is transferred into the resist with a defined gradient (typically linear) along one coordinate. In the present study, a linear dose gradient was employed. After exposure and development, the profile of the formed structure in the resist is measured using atomic force microscopy (AFM), which enables the determination of the residual resist thickness along the wedge. The resulting spatial profile is then converted into a dose scale, yielding the dose curve.

Since obtaining a single dose–response curve that spans a wide range of exposure doses (especially with a constant dose step size) is technically challenging, the exposure process is practically implemented as a series of dose wedges, each covering a limited but partially overlapping dose range. Depending on the specific objectives of the study and the required precision, exposures in different dose intervals may be carried out using varying dose step sizes. The resulting segments of the dose–response curves, which typically exhibit overlapping regions, are subsequently merged into a single composite curve that encompasses the entire range of interest. For clarity, the final dose–response curve is typically presented on a semi-logarithmic scale. It is shown in Figures 1 and 2a of the manuscript.

Design and AFM Profiling of the Dose Wedge Structure

In the first part of the experimental study, a thin PMMA 950K resist film with a thickness of approximately 50 nm, spin-coated onto a silicon substrate, was investigated to evaluate its sensitivity to gallium ion exposure at an energy of 30 keV. The ion beam exposure was carried out at a beam current of 10 pA; these parameters were kept constant for all structures, while the dose variation was achieved by adjusting the exposure time. To cover a broad dose range and to analyze both the positive-tone and negative-tone behavior of the resist, several dose wedges were employed, each designed with a specific dose range and step size. The parameters of the dose wedges used in the study are summarized in Table S1 (Supplementary Materials).

Typical dose wedges corresponding to the positive- and negative-tone behavior of the resist are shown in Figure S1 (Supplementary Materials). The total length of the dose wedge for ion beam exposure was

chosen based on the horizontal field width (HFW) of the AFM scan area. A wedge length of $L = 55$ µm was used, which, for a ten-step pattern, resulted in an individual cell size of approximately 5.5 µm.

Table S1. Parameters of the dose wedges used for sensitivity and tone behavior analysis under 30 keV Ga^{+} ion exposure.

Dose Wedge No.	Dose Range (µC/cm²)	Dose Step (µC/cm²)
1	0 – 0.4	0.04
2	0 – 1.0	0.1
3	0 – 3.0	0.3
4	0 – 10.0	1.0

Figures S1a and S1c present AFM images of the dose wedges (positive- and negative-tone) with a marked line indicating the direction of the linear scan used to construct the spatial depth profile. The corresponding profiles are shown in Figures S1b and S1d (positive- and negative-tone). These profiles were linearly converted into exposure dose values based on the parameters listed in *Table S1* and merged into a single dose curve. The curve is plotted as normalized resist thickness versus exposure dose on a logarithmic scale and is presented in the main text of the manuscript in Figure 1.

Positive-Tone Resist Behavior (for doses below ~0.5 µC/cm²)

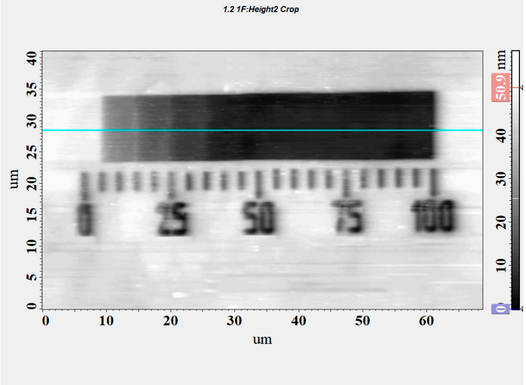

(a)

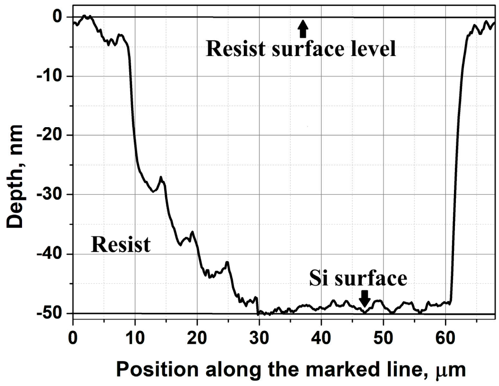

(b)

Negative-Tone Resist Behavior (for doses above ~1 µC/cm²)

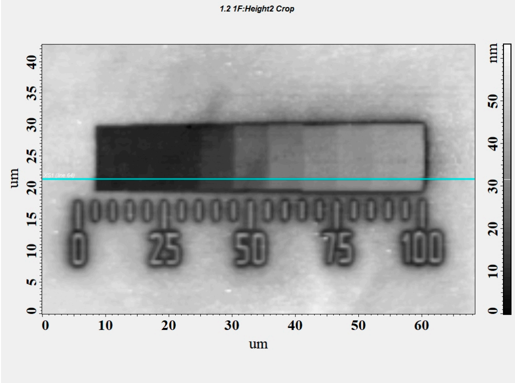

(c)

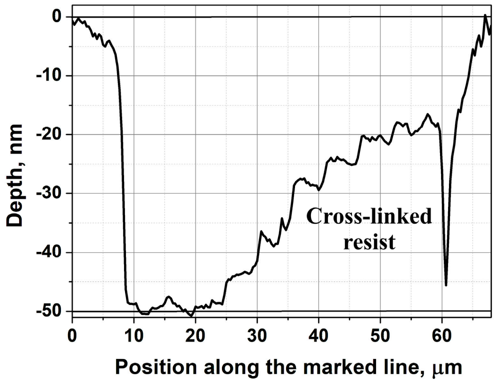

(d)

Figure S1. Typical dose wedges #2 and #3 used to characterize positive- and negative-tone behavior of a 50 nm PMMA 950K resist layer on a silicon substrate under 30 keV Ga^+ ion exposure.

Electron beam exposure was carried out on the same sample (Si wafer with the resist layer) that had previously been used for ion beam exposure, using an accelerating voltage of 30 keV and a beam current of 30 pA. After exposure, this sample was developed simultaneously with the dose wedges patterned by the ion beam lithography, ensuring a consistent basis for comparing sensitivity and contrast. The use of the same sample guaranteed identical resist thickness, as well as the same thermal processing and development conditions for both exposure types.

To cover a broad dose range and to investigate both the positive-tone and negative-tone behavior of the resist, dose wedges with different dose ranges and step sizes were employed. The total length of the dose wedge for electron beam exposure was also selected based on the horizontal field width (HFW) of the AFM scan area but was slightly longer: the wedge length was L = 60 μm, which, for twenty steps, resulted in an individual cell size of approximately 3 μm. The parameters of the dose wedges used in the electron beam exposure study are summarized in Table S2 (Supplementary Materials).

Table S2. Parameters of the dose wedges used for electron beam exposure to evaluate resist sensitivity, contrast, and tone behavior.

**Dose Wedge No.**	**Dose Range (μC/cm^2)**	**Dose Step (μC/cm^2)**
1	0 – 100	5
2	0 – 4 000	200
3	0 – 10 000	500

In the second part of the experimental study, the analysis of the development depth profile was carried out on a thick PMMA 950K resist layer with a thickness of 180 nm. All exposure parameters, including the characteristics of the gallium ion beam and the configuration of the dose wedges, were kept identical to those used in the first stage, where the sensitivity and tone behavior of the resist were investigated. The depth profile was obtained by sequentially merging data from four individual dose ranges listed in Table S1. Figure S2 presents the step-by-step process of stitching these profiles together, covering the full dose range from 0 to 10 μC/cm^2. In Figure S2a, the original linear AFM depth profile for dose wedge #4 is shown. In Figure S2b, this same profile is linearly converted into dose units along the scan axis. Figure S2c shows all individual profiles — #1, #2, #3, and #4 — combined in a single graph plotted in the conventional semi-logarithmic scale. Finally, Figure S2d presents the full stitched depth–dose profile across the entire range, which is also shown in the main manuscript as Figure 2a.

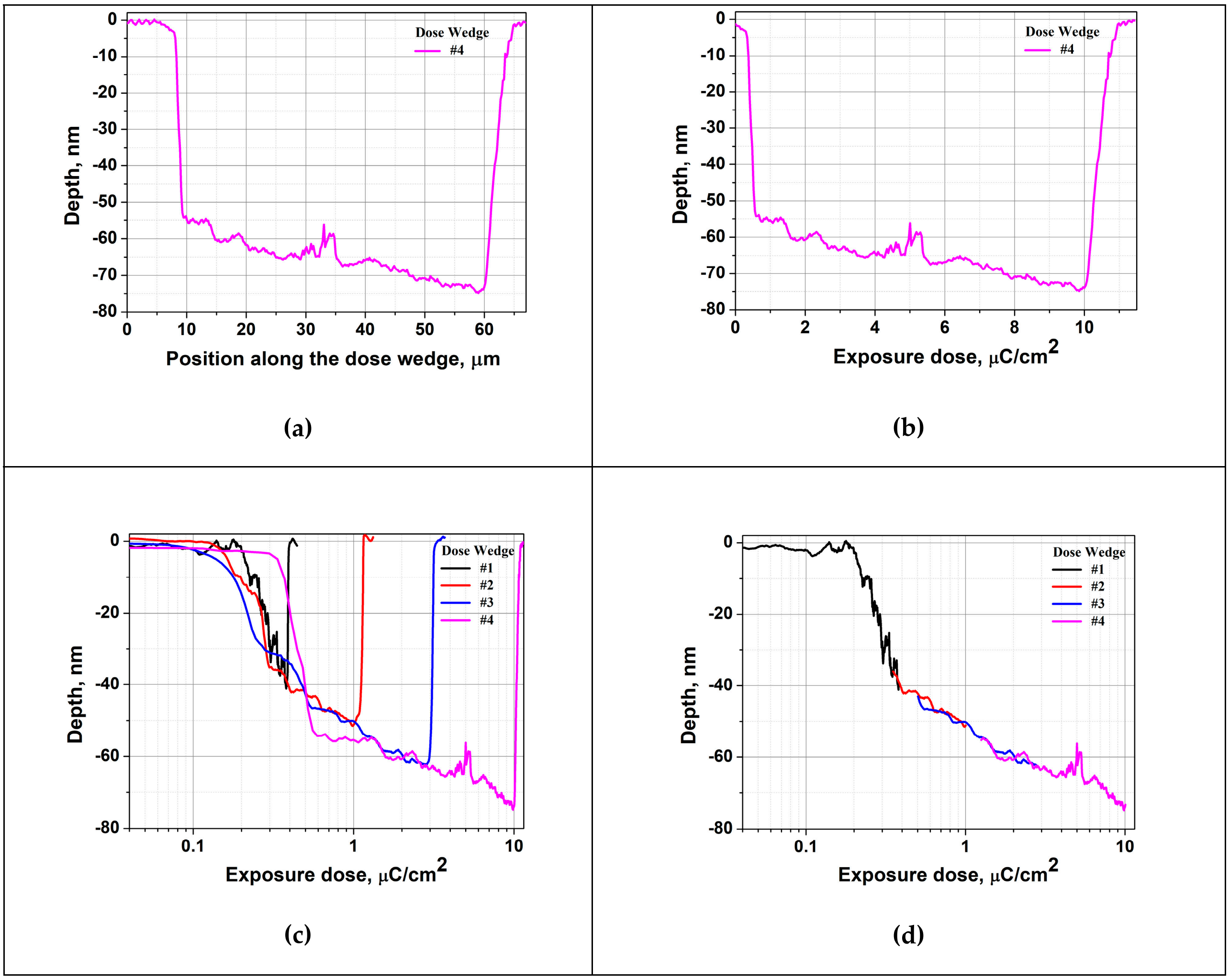

Figure S2. Construction of the full depth–dose profile in a 180 nm PMMA 950K resist layer on a silicon substrate under 30 keV Ga^+ ion exposure: (a) AFM profile of wedge #4; (b) dose conversion; (c) merged wedges #1–#4; (d) final stitched curve.
